# Practical Notes on Diagnosis and Treatment

**Published:** 1907-01-12

**Authors:** 


### Practical Notes on Diagnosis and Treatment.

**The Value of Inspection.**

It is an important rule to look well at a diseased or injured part before manipulating it.—*Mr. Thos. Bryant.*

**Pilocarpine in Threatened Mania.**

In cases showing much mental excitement, and where such soporifics as opium and chloral have been known to fail, a hypodermic injection of nitrate of pilocarpine is often most successful.

**Phthisis and Morbus Cordis.**

The prognosis of phthisis associated with morbus cordis is always good as to duration except in the rare cases in which the endocarditis takes on the malignant form. It is well known that the association of these diseases is uncommon.—*Dr. Percy Kidd.*

**Chlorate of Sodium in Cancer.**

Some few years ago Dr. Brissaud, of Paris, reported several cases of malignant disease of the stomach treated with sodium chlorate; the dose varied from 2 to 4 drams in the 24 hours. He claimed very remarkable results in cases in which the disease had not extended to the liver or other organs.

**Iodised Collodion for Ringworm.**

The following is recommended:—Dissolve 10 grains of iodine in 3 drachms of rectified spirit, add 1½ oz. of collodion, Venice turpentine 24 grains, and castor-oil ½ drachm. Apply to the affected area for 3 or four days, so as to cover with a thick adherent layer. Peel off in a fortnight's time, and wash the surface with solution of mercury perchloride (1 in 500).

**Opium in Elderly Patients.**

In elderly patients who are suffering from chronic ailments and general weakness of health, doses of opium given systematically at bed-time have a most beneficial effect, not merely in procuring sleep, but in acting as a tonic. Five grains of soap pill given at night, or even twice a day, will make these patients very much more comfortable and happy than any amount of stimulant.—*Mr. Christopher Heath.*

**Hæmatemesis v. Hæmoptysis.**

Why it should be I cannot quite explain, but, clinically, it is the case that a moderate amount of blood from the stomach is far more likely to be accompanied with faintness, vertigo, or dizziness than the same amount from the lungs, provided, of course, the quantity lost is not so excessive as would induce a state of syncope, whatever its source.—*Dr. Allchin.*

**Nitro-Glycerine in Epilepsy.**

Dr. Elliott Bates has used nitroglycerine by hypodermic injection in cases of epilepsy. The dose employed was $\frac{1}{100}$ grain. The stage at which the medicine is employed is immediately after the establishment of the attack and when the patient is lying in a state of unconsciousness. It is claimed that the treatment secures prompt return of consciousness. Further, the after-effects of the attack are much diminished in severity.

**Paracentesis in Ascites.**

In hepatic dropsy as soon as there is enough ascites to make you sure of not wounding the intestines it is time to tap. Drugs will act much better afterwards.—*Dr. P. H. Pye-Smith.*

**Nocturnal Enuresis.**

One method recommended in the treatment of this condition, in the case of boys, is to close the preputial orifice by applying collodion when the patient goes to bed. The layer closing the meatus can readily be removed with the finger when the patient wishes to micturate.

**Tepid Sponging in Insomnia.**

I am often tempted to think that those who rush to antipyrin, sulphonal, phenacetin, etc., for the reduction of temperature, or for the production of sleep, have too little experience of the antipyretic and sedative effects of tepid sponging of the face, chest and neck, especially the carotid regions of the latter.—*Dr. F. J. Smith.*

**Significance of the Cracked Pot Sound.**

Though it may often be obtained in phthisis pulmonalis this sound is not pathognomonic of that disease. It may be present in pneumonia and even in the upper part of the lung when the base is compressed by a pleural effusion. In the case of children it may be elicited over a perfectly healthy lung.

**Treatment of Hæmorrhoids.**

In patients who live a sedentary life adequate exercise will often cure piles. A useful local application for hæmorrhoids which present externally is: Morphine 10 grs., tannic acid and extract of belladonna, of each 1 dram, lanoline and vaseline, of each 1 ounce. In internal piles, a good injection is sulphate of iron, 10 grs., in 1 ounce of water, injected warm after the bowels have acted.

**Stimulation of Medulla Oblongata.**

The measure that has seemed to me to have most influence in stimulating and keeping up the functions of the medulla, is the hypodermic injection of a small quantity of strychnine, that is one-eightieth of a grain, together with a very minute, stimulant dose of morphine, about one thirty-sixth of a grain, and this repeated every two hours, if necessary.—*Sir Wm. Gowers.*

**Treatment of Seborrhœa.**

The only thing which permanently succeeds in stopping this affection is absolute disinfection or sterilisation of the scalp. For this purpose the scalp should be treated for a few days with perchloride solution 1 in 1,000-2,000; an antiseptic soap may be used. Then, the next remedy to apply is sulphur, which has a most definite effect. What I generally prescribe is precipitated sulphur 15 grs., carbolic acid 15 min., vaseline 1 ounce. To this may be added a few drops of oil of lavender or oil of bergamot. The antiseptic washing should be repeated from time to time, say, once a week, and brushes, combs, hat-linings, etc., should also receive antiseptic treatment.—*Dr. Payne.*

